# Correction: ToxoNet: A high confidence map of protein-protein interactions in *Toxoplasma gondii*

**DOI:** 10.1371/journal.pcbi.1014086

**Published:** 2026-03-13

**Authors:** Lakshmipuram S. Swapna, Grant C. Stevens, Aline Sardinha-Silva, Lucas ZhongMing Hu, Verena Brand, Daniel D. Fusca, Cuihong Wan, Xuejian Xiong, Jon P. Boyle, Michael E. Grigg, Andrew Emili, John Parkinson

The fourth author’s name is spelled incorrectly. The correct name is: Lucas ZhongMing Hu. The correct citation is: Swapna LS, Stevens GC, Sardinha-Silva A, Hu LZ, Brand V, Fusca DD, et al. (2024) ToxoNet: A high confidence map of protein-protein interactions in Toxoplasma gondii. PLoS Comput Biol 20(6): e1012208. https://doi.org/10.1371/journal.pcbi.1012208
