# Supplementary figures and images for: ToxoNet: A high confidence map of protein-protein interactions in Toxoplasma gondii
Source: PLoS Comput Biol. 2024 Jun 20;20(6):e1012208. doi: 10.1371/journal.pcbi.1012208 (PMC11219001; doi:10.1371/journal.pcbi.1012208)

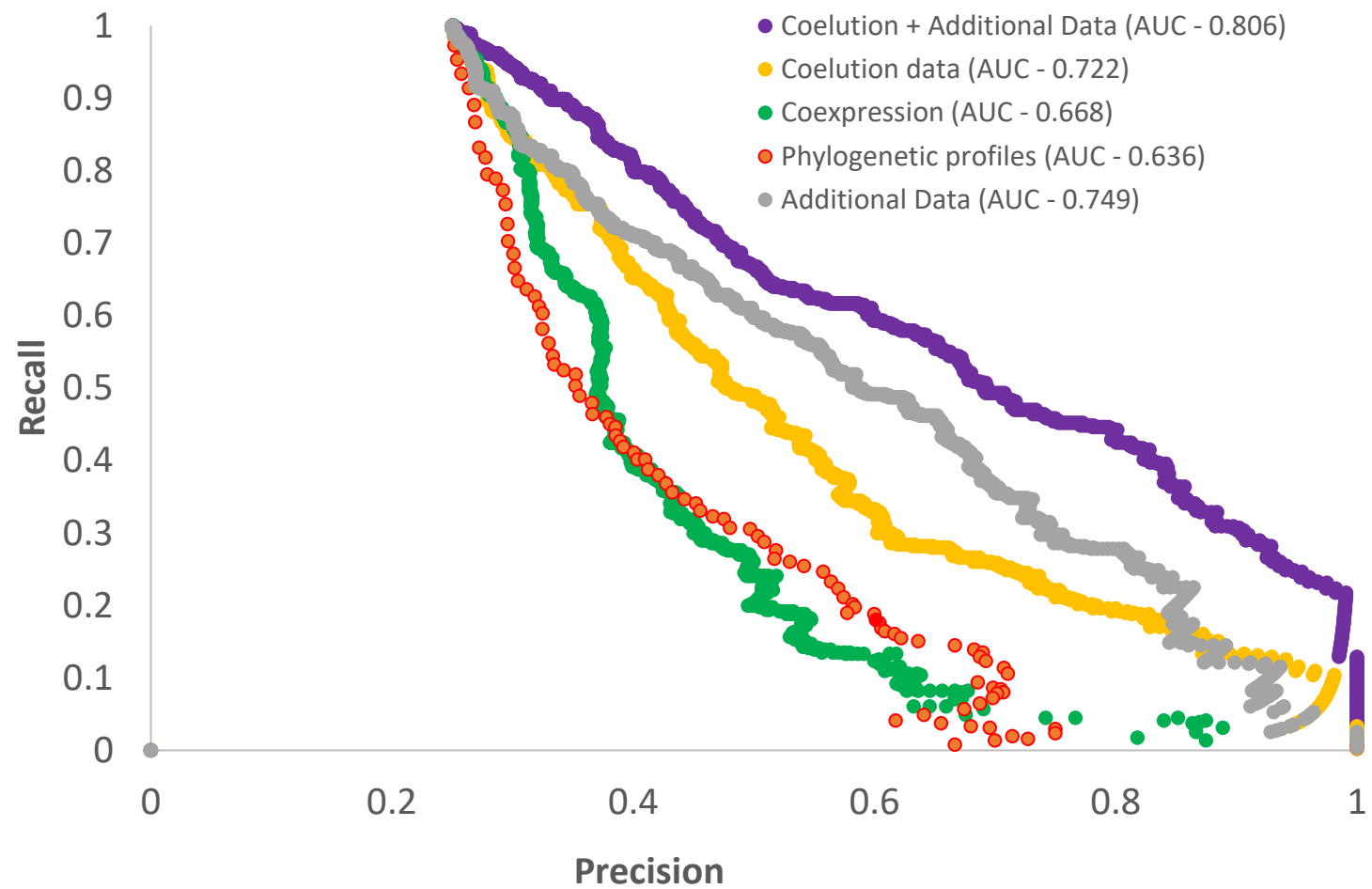

Supplement: S1 Fig — (PDF) [file pcbi.1012208.s001.pdf]

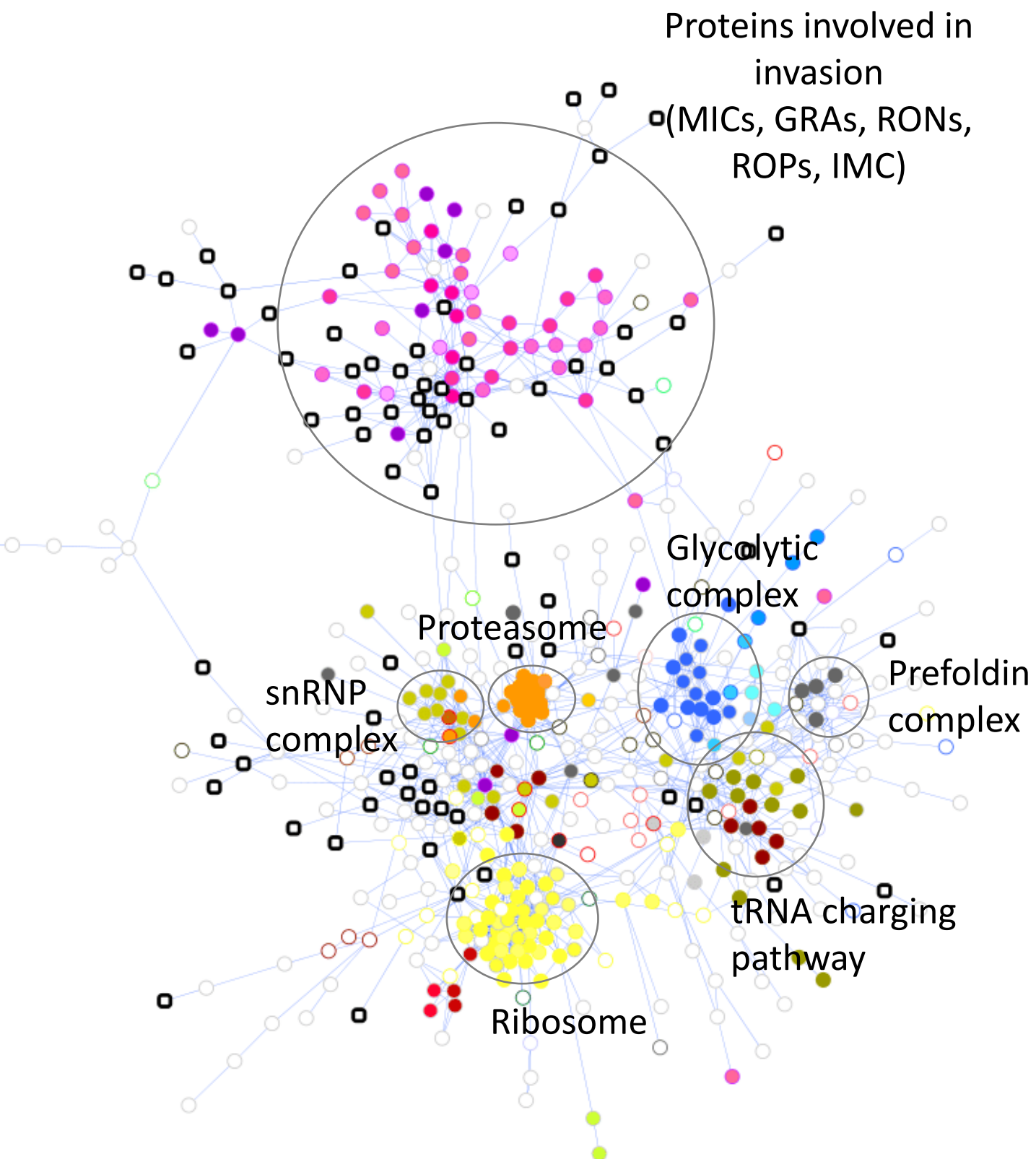

Supplement: S2 Fig — Hypothetical proteins are indicated as squares enclosed by black borders. Well known protein complexes are colored uniquely and encircled–such as ribosome (yellow), proteasome (orange), snRNP complex (light green), glycolytic complex (blue), prefoldin complex (black). Proteins known to be involved in invasion are colored pink, and proteins associated with the IMC are colored purple. (PDF) [file pcbi.1012208.s002.pdf]

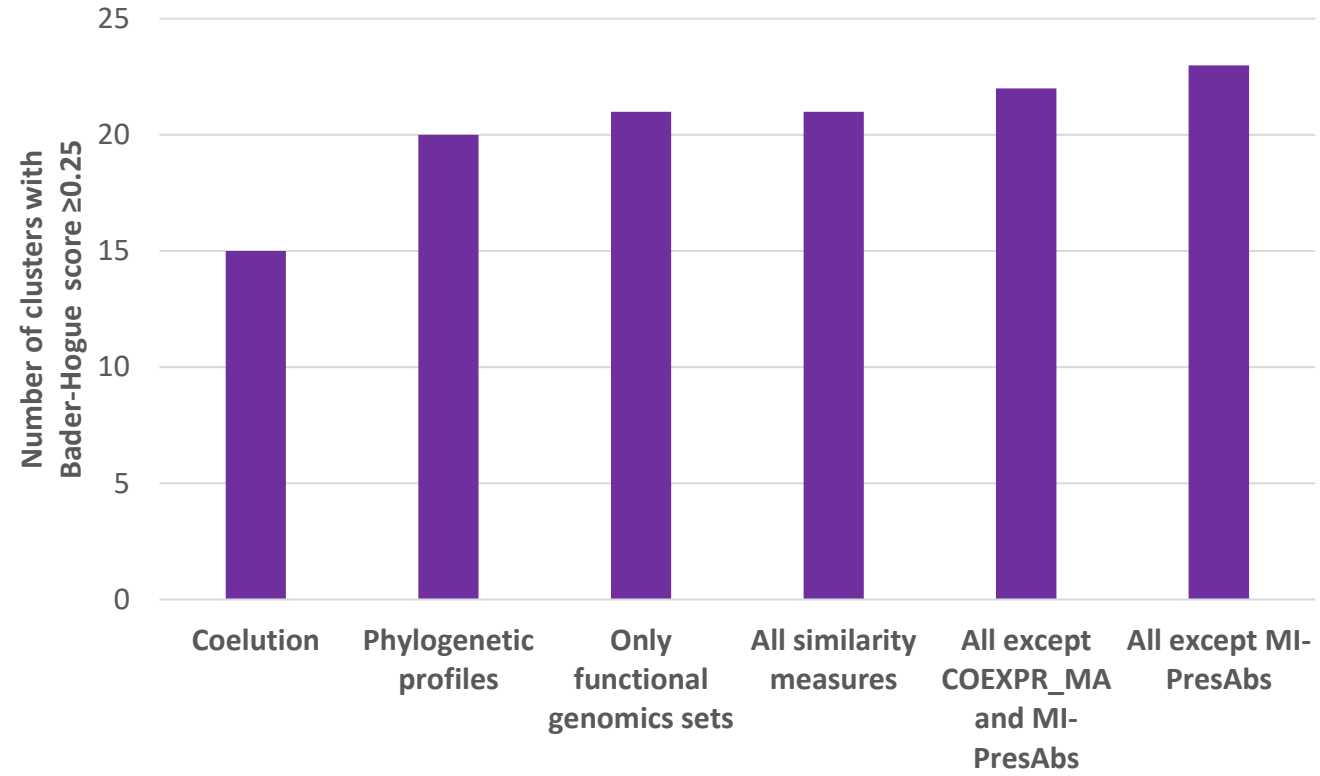

Supplement: S3 Fig — After network generation, clusters were defined using the MCL algorithm and evaluated for overlap with known protein complexes using the Bader-Hogue overlap scoring algorithm. The graph shows the number of unique clusters with overlap score ≥0.25 with respect to known protein complexes for six combinations of networks: 1) coelution scores (PCCNM, WCC and Coapex1); 2) phylogenetic scores (MI_PresAbs, MI_pij); 3) functional genomics scores (MI_PresAbs, MI_pij, COEXPR_RS and COEXPR_MA); 4) coelution and functional genomics scores (PCCNM, WCC, Coapex1, MI_PresAbs, MI_pij, COEXPR_RS and COEXPR_MA); 5) coelution and functional genomics scores excluding COEXPR_MA and MI_PresAbs; and 6) coelution and functional genomics scores excluding MI_PresAbs. In this analysis, phylogenetic profile scores performed better than coelution scores. However, overall we found the network generated from combining coelution and functional genomics scores, excluding MI_PresAbs, gave the best performance. This network was selected as the final unsupervised network. (PDF) [file pcbi.1012208.s003.pdf]

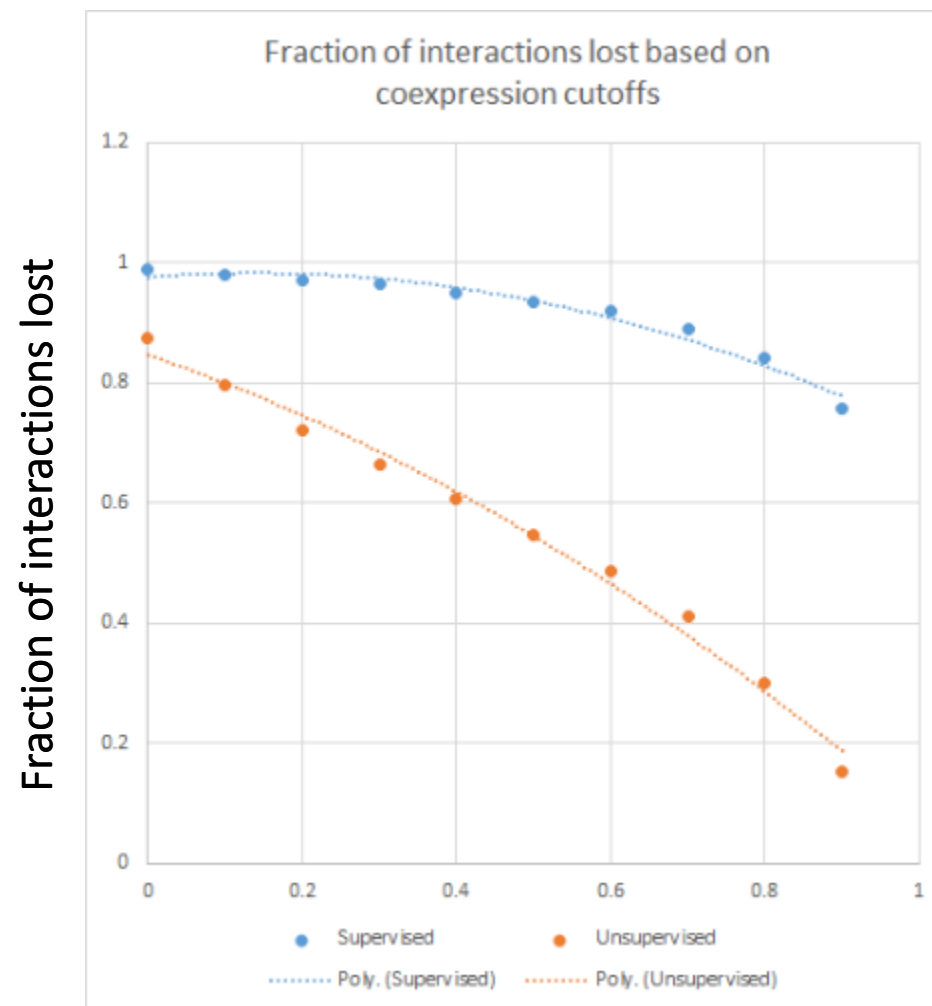

Coexpression cutoffs (based on pearson correlation coefficient)

Supplement: S4 Fig — (PDF) [file pcbi.1012208.s004.pdf]

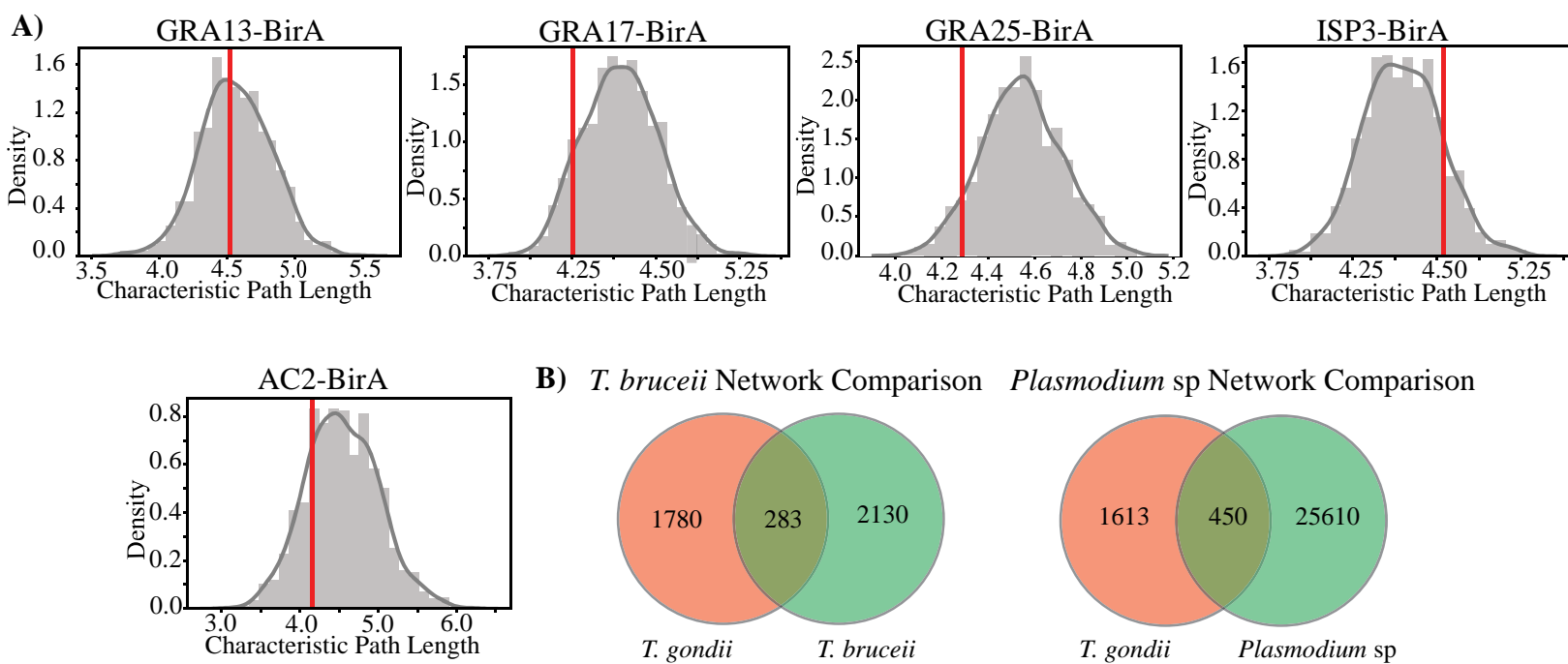

**B) *T. brucei* Network Comparison    *Plasmodium* sp Network Comparison**

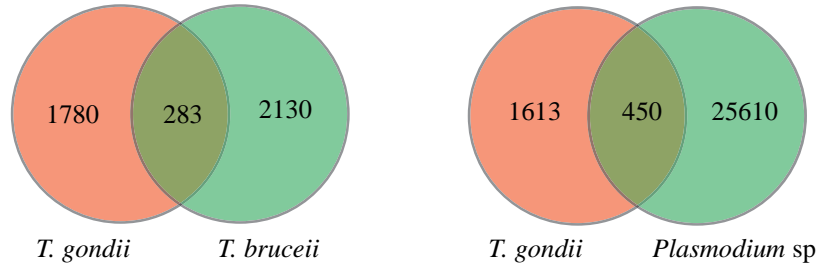

Supplement: S6 Fig — (A) The distribution of random permutations (n = 1000) of characteristic path lengths relative to the actual characteristic path length (red line) of network proteins identified in biotinylation BirA-based BioID experiments with GRA13 (n = 35, p = 0.43), GRA17 (n = 48, p = 0.1), GRA25 (n = 71, p = 0.07), ISP3 (n = 46, p = 0.85), AC2 (n = 14, p = 0.21). (B) The intersection of interactions predicted in recent Trypanosome bruceii6 and Plasmodium falciparum7 protein interaction networks. (PDF) [file pcbi.1012208.s006.pdf]

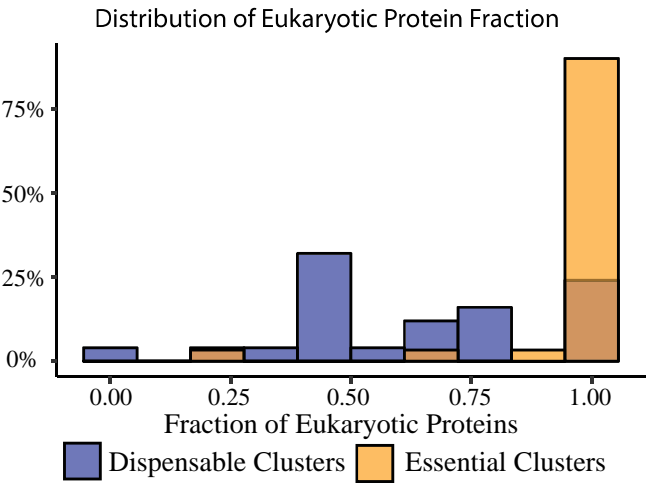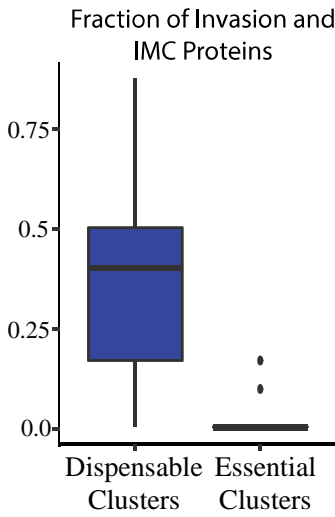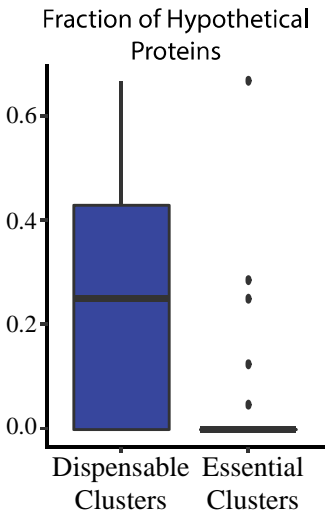

Supplement: S7 Fig — (a) Overlapping histograms compare the distribution of the fraction of conserved eukaryotic proteins in essential and dispensable clusters. Boxplots illustrate the fraction of invasion and IMC proteins (b) and hypothetical proteins (c) in essential and dispensable clusters. (PDF) [file pcbi.1012208.s007.pdf]

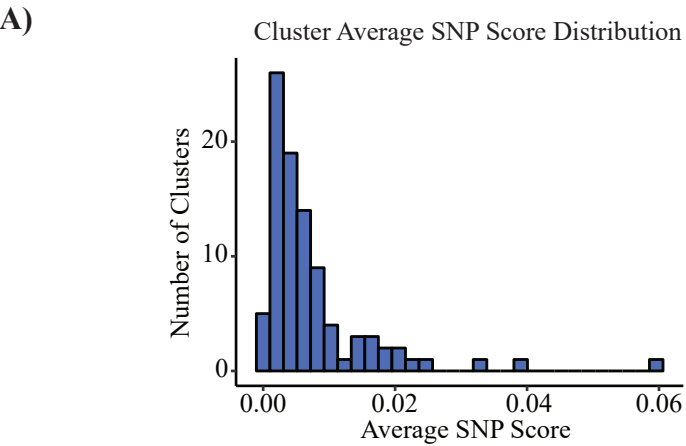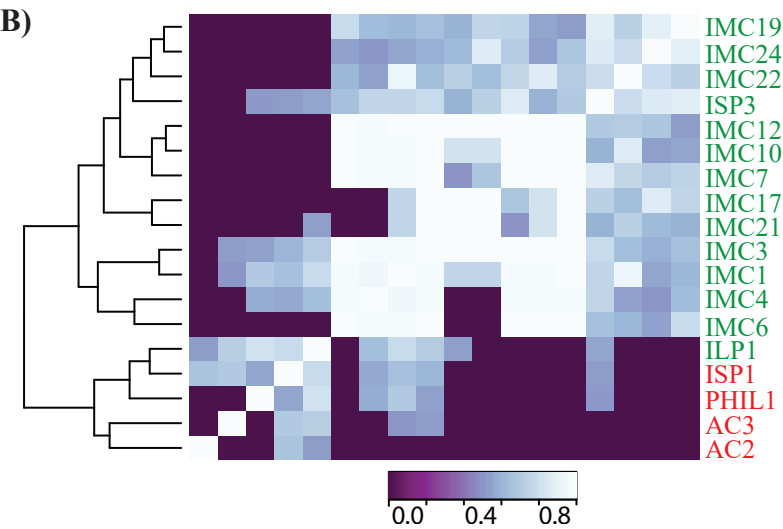

Supplement: S8 Fig — (A) Distribution of the average SNP score for each predicted protein complex. (B) Hierarchal clustering of the best pairwise WCC score from each coleution experiment for IMC proteins recapitulates the subcompartmental structural organization of the IMC. Proteins previously localized to the apical and basal/central subcompartments are highlighted in red and green, respectively. (PDF) [file pcbi.1012208.s008.pdf]
