# Supplementary material for: ToxoNet: A high confidence map of protein-protein interactions in Toxoplasma gondii
Source: PLoS Comput Biol. 2024 Jun 20;20(6):e1012208. doi: 10.1371/journal.pcbi.1012208 (PMC11219001; doi:10.1371/journal.pcbi.1012208)

A) Distribution of proteins in low confidence network

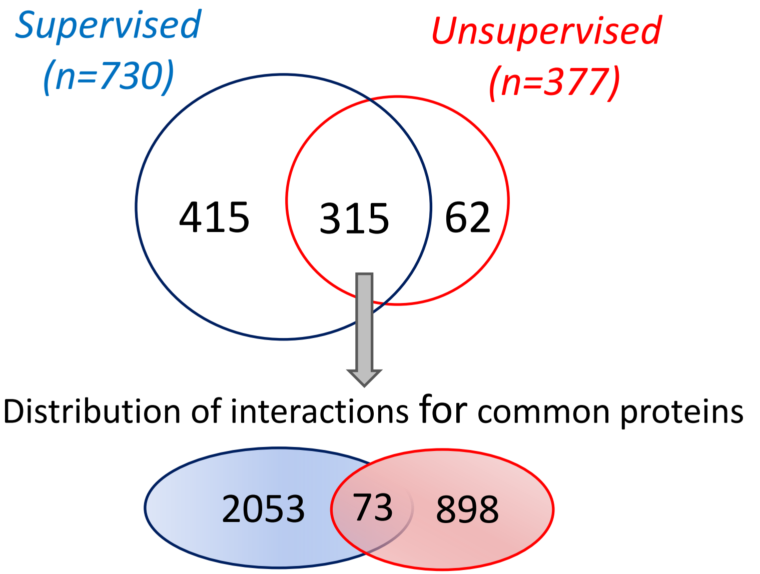

B) Distribution of spectral counts

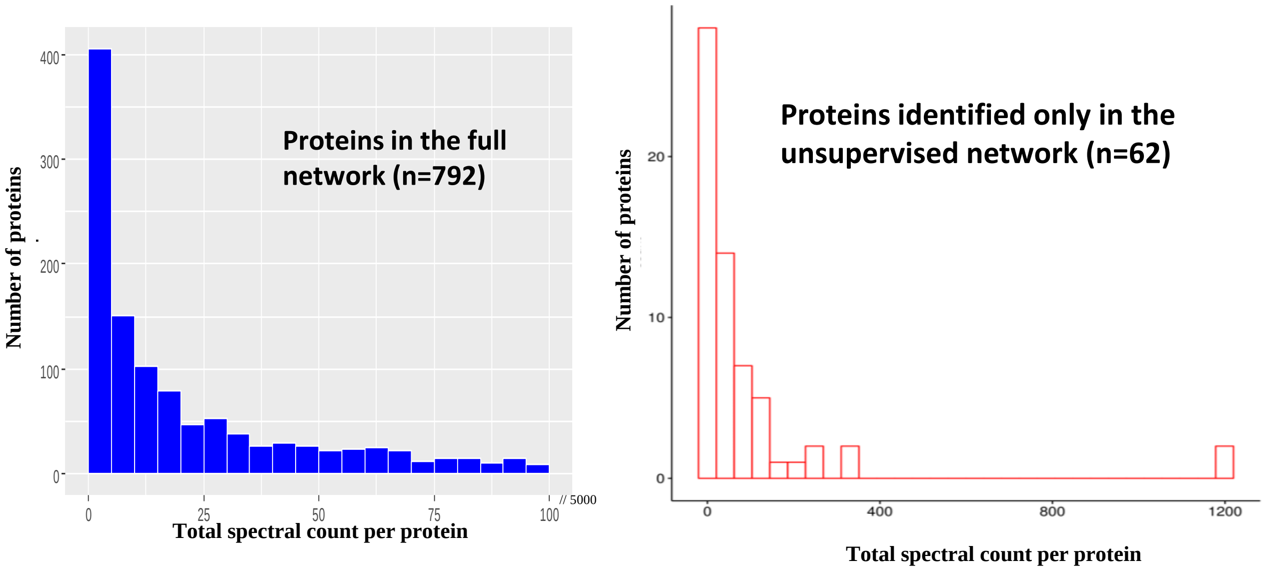

C) Distribution of scores for Supervised vs. Unsupervised vs. Random Interactions

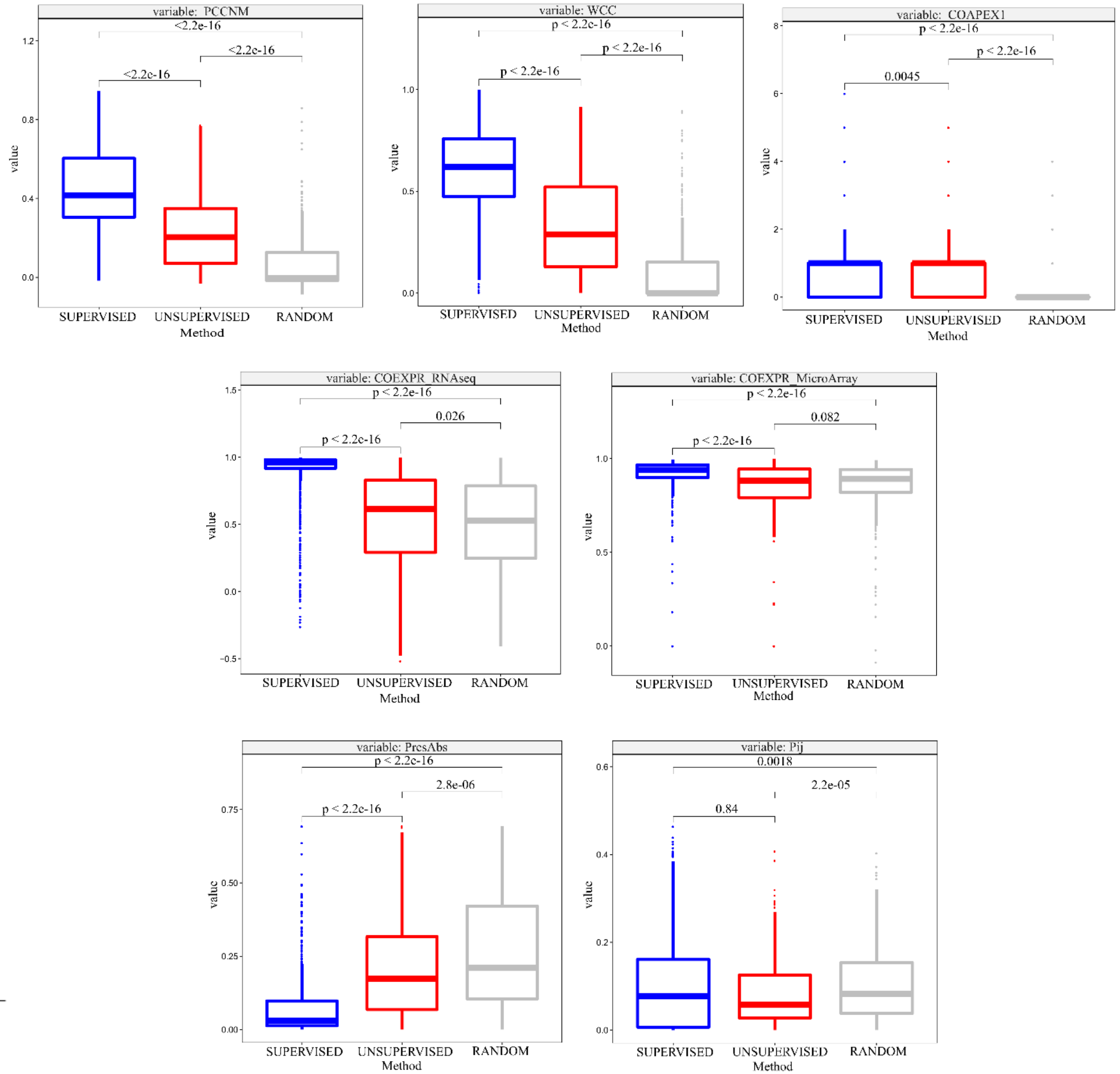

Supplement: S5 Fig — (A). Venn diagram showing the number of overlapping and unique proteins for the supervised and unsupervised networks. (B) Features of proteins uniquely identified by the unsupervised network: Distribution of spectral counts for the proteins, Box plot comparing the PCCNM scores for the interactions of these proteins with an equivalent set of randomly generated interactions (C). Box plots depicting the distribution of various coelution, coexpression, and phylogenetic scores for Supervised, Unsupervised, and an equivalent set of Randomly generated interactions. The box and whiskers in each boxplot indicate the 25%-75% quartile and min-max of the scores over all the interactions in a dataset, respectively. (PDF) [file pcbi.1012208.s005.pdf]
