# Supplementary material for: ToxoNet: A high confidence map of protein-protein interactions in Toxoplasma gondii
Source: PLoS Comput Biol. 2024 Jun 20;20(6):e1012208. doi: 10.1371/journal.pcbi.1012208 (PMC11219001; doi:10.1371/journal.pcbi.1012208)
Supplement: Attachment — Submitted filename: 240210_Review.docx [file pcbi.1012208.s019.docx]

Review for Swapna et al, ToxoNet. Plos Comp Biol.

In this work Swapna et al use a combination of supervised and unsupervised machine learning methodologies to integrate experimental data (biochemical co-fractionation) with functional genomics annotations (co-expression, phylogenetic profiling and domain interactions) to construct a map of protein-protein interactions in Toxoplasma gondii. The approach of combining both supervised and unsupervised methods is interesting and well-suited to a non-model organism that has limited “gold standard” training data. In general the methods are appropriate, however the rigor of the supervised model training and subsequent evaluation needs improvement. The methods is lacking some information that is necessary to understand what was done. Additionally, improving the precision of language throughout the manuscript is necessary to make it easier to follow the process of model training and data integration.

Major Comments/concerns:

1. Rigor of the supervised model training. The standard approach in machine learning is to split a set of “gold standard” data into training and test (validation) sets. The model is trained (often with cross validation) on the training set and then evaluated on a test set that was *not* included during training. As best I can tell, here this does not appear to be the case. My understanding is that the supervised PPI network was both trained and evaluated on a single training set. If this was the case, the model should be evaluated on a separate data set that was not included during training to have a more “fair” measure of performance. A few follow-on questions might help clarify this:
   a. In figure 1, and supplementary figure 1, what data are included in the ROC and precision/recall curves that are used for network evaluation? Are these for the full training data set, or do they indicate an average performance across the 10 cross validation runs? Or were these computed on a separate test set that was not included during training?
   b. Similarly, is the AUC of 0.806 reported in the methods (based on 10-fold cross validation) correspond to the performance across cross-validation sets, or across all the training data or across a separate hold out test set?
2. Model performance given inclusion of different scoring information (features). It is not clear to me how much information the experimental co-fractionation data add above the functional genomics annotations. While figure 1 and supplemental figure 1 compare the “co-elution + additional data curve” to a supervised model constructed with either co-expression or phylogenetic profiles, there is notably not a comparison to a supervised random forest classifier trained on ALL of the functional genomics data (co-expression, phylo profiles, domain-domain interactions and STRINGdb).
3. Methodological information. It would help to have more clarification on the process used to train the supervised model. In particular, what hyperparameters were tuned or optimized when training the Random Forest Classifier (maximum tree depth, minimum samples to split a node, others)? Also, how were the hyperparameters assigned following the 10-fold cross validation (did they take the average across the 10 training sets, or iteratively update the parameters, or something different?)
4. Figure 1 panel A – To me, it seems that the scores (“features”) included during model training are inconsistent between this figure panel and what is listed in Supplemental Table 3. The figure suggests that 3 co-elution similarity scores were included during supervised random forest training, but from the main text and supplement it seems to me like 45 different measures of co-elution similarity were computed?

Minor Comments:

1. Precision of language – “scores”. Throughout the manuscript the authors refer to scores, which sometimes mean correlative measures drawn from experimental data, and sometimes mean measures drawn from functional genomics data. The functional genomics data are sometimes (though not always) called “additional data”. It would help the reader to use more descriptive, consistent naming for the scores (e.g. coelution similarity scores, functional genomics scores) throughout the paper to make it absolutely clear what is being used in the model.
2. Precision of language – model naming schemes. In some places the network constructed using the supervised and unsupervised approaches is called the “full network” and in other places “the combined network”. It would help the reader to use only one term. In my opinion “combined network” better captures the idea that the model is using information from the supervised and unsupervised approaches.
3. It is not also not always clear when the authors are referring to “the combined network” and the “combined high confidence network” – for example, the introduction (p.6) states that the “pipeline yielded a network of 3,753 interactions capturing 792 proteins” while the abstract says “2,063 interactions connecting 652 proteins”. When I read this the first time I was confused by the difference in the two sets of numbers. It would be good to indicate (already in the intro) that the larger network was filtered to obtain the numbers in the abstract, and start consistently referring to the networks as the “combined model” and “combined high confidence model” from the beginning.
4. On p.8 the authors introduce the “scoring schemes” (“These scoring schemes were calculated individually for the 6 experiments and then once more…”). Here it would be useful to indicate that there are a total of 45 coelution correlation scores computed to give the reader a sense of the number of features.
5. Also on p.8 the authors should mention that several measures from STRING db were included as features (“functional genomics scores”) even though they did not add information (according to the red cells in table S3). At present, one needs to look in detail at the methods/supplement to learn that these were considered but did not add information.
6. The size of the test set is described in the methods, but is sufficiently important to evaluate the results that it should be promoted to the main text (perhaps near where supplemental table 4 is introduced).
7. On page 9, I am confused about the “individual networks” that were constructed for the Similarity Network Fusion. Did the authors construct 7 individual networks, each based on one of the 7 scores listed (e.g. one PCCNM network, one WCC network, etc), or were the 7 networks each based on a different combination of the 7 scores (e.g. a mix of PCCNM, COEXPR-MA and MI-PresAbs)? Can the authors provide more insight into the rationale for which measures were combined/used to make the 7 individual networks?
8. Start of paragraph 2 on p 19 – “We exhaustively explored all possible combinations of parameters”. What parameters are being referred to here? Are these combinations of scores used to construct the networks, or model hyperparameters or something else? What was the size of the possible parameter space?
9. On p 10, last paragraph “Comparison of the overall supervised and unsupervised networks” – Here is the comparison between the unfiltered networks, or the filtered high-confidence networks? Again, having specific names for each network that are used consistently throughout would be a big help.
10. Very minor – p. 11 “(pcc-noise model, weighted cross correlation, overall coapex score 1)” – here the authors deviate from the abbreviations used elsewhere in the paper (PCCNM, WCC)
11. p.11 “however, they are worse than that of supervised scores for PCCNM and WCC…” – I am not sure here what is being compared. Do you mean that the predicted pairwise interactions from the unsupervised model have PCCNM and WCC scores that are lower than predicted interactions in the overall supervised tree? Or do you mean that the scores of the predicted interactions from the unsupervised model are systematically lower than top hits in supervised models constructed with *only* PCCNM and WCC scores? Please define more carefully what is being compared.
12. p.11 “Based on the six scoring schemes outlined above” – I think the authors mean “six co-elution similarity scores outlined above” but the wording is somewhat ambiguous.
13. Methods section “Capturing similarity of co-elution profiles” – p. 32. Potential typo: “Calculate similarities over 420 fractions (PCCNM_overall…)”. Within the parenthetical that lists the scoring schemes both MI-overall and MI_overall are listed, but Coapex_X is excluded?
14. Methods section “Supervised machine learning approach” – p. 35. “61 features in total comprising of both coelution and functional dataset..” I am confused about how there are 61 features. From looking at Supplemental Table 3 I think there are 52 total features (45 coelution similarity scores and 7 functional genomics scores)? Are there additional features that I am missing?
15. Methods – p. 36 “each of the permutations were clustered using MCL” – please define the abbreviation.
16. Methods – p. 37 “For the supervised network, the network generated based on feature selection was chosen” – I am confused about what this means. Were other networks generated that were *not* based on feature selection? Or do the authors mean that they took the most informative features (colored green in supplemental table 3) and then retrained a new network after eliminating the non-informative features?
