# Supplementary material for: ToxoNet: A high confidence map of protein-protein interactions in Toxoplasma gondii
Source: PLoS Comput Biol. 2024 Jun 20;20(6):e1012208. doi: 10.1371/journal.pcbi.1012208 (PMC11219001; doi:10.1371/journal.pcbi.1012208)
Supplement: Attachment — Submitted filename: Response_220424.docx [file pcbi.1012208.s020.docx]

We would like to thank the editor and reviewers for their time and efforts in carefully appraising our manuscript. Below we provide a point-by-point response to the concerns raised during the review process. For changes to the main manuscript, we reference **line numbers where changes have been made which refer to the MARKED UP** (i.e. NOT clean) copy of the manuscript. In addition, during revision of the manuscript we noted a recent study had cited our dataset in support of a novel tetrametric complex confirming our predictions of interactions between GRA32 (TGME49_212300), GRA70 (TGME49_249990) and GRA71 (TGME49_309600) and further supporting the value of this dataset [1]. We now reference this study in the revision (Lines 632-635).

*Editor:*

*Please pay close attention to comments regarding the issue of the apparent low correlation between hyperLOPIT predicted localization in identified complexes (i.e. many complexes have components that are apparently in different compartments).*

As requested we have taken this comment very seriously and provide detailed responses to specific reviewers comments below regarding the correlation with hyperLOPIT predicted localizations.

*Reviewer #1:*

*Toxonet is an excellent resource for the toxoplasma community, combined with some interesting analysis I think this is a great paper. It includes new data, new appproaches and follow up analysis in several cases.*

We would like to thank the reviewer for their time and effort in providing their feedback and overall enthusiasm for our study. *I have some minor comments that I hope that authors can address.

1) Could the authors, where possible, show the pointers underlying the data - i.e. when they present error bars?*

As requested we now provide the datapoints in the appropriate figures (Figures 2C-2E).

*2) The authors show results improve with adding more data quite convincingly but how SNF compares with other approaches for data integration is not clearly presented in the manuscript.*

We apologize for the lack of clarity regarding the performance of the SNF algorithm. In this study our main focus was on generating a high-confidence set of protein-protein interactions for *Toxoplasma gondii*. To offset the relative lack of gold-standard interactions to use for training supervised machine learning approaches, we compared the usage of a supervised approach (here RandomForest) with a non-supervised approach (here SNF). We show that the two approaches are complementary (**Supplementary Figures 5A and B**). We also found that while the predictions generated using SNF are not of the quality observed from RandomForest, they are significantly better than random (**Supplementary Figure 5C** – note lower mutual information scores for PresAbs and Pij indicate the profiles for the two proteins are more similar). In the revised manuscript, we also include a figure which investigates how the inclusion of co-elution and functional genomics datasets contributes to the quality of networks generated by SNF in terms of protein clusters (as measured by the Bader-Hogue score - **Supplementary Figure 3**). This shows that by themselves, phylogenetic profile similarity scores offer improved performance relative to coelution scores. However, integration of all scores (with the exception of MI_PresAbs – see below) resulted in the best performance in terms of the number of predicted clusters that overlap with known protein complexes. This network was selected for downstream analyses. These details have been added to the main text in Results, Methods and Supplemental Legends (Lines 229-235, 870-903 and 1641-1655).

While this work shows for the first time that a non-supervised data integration approach can serve as a useful framework for predicting protein interactions, we agree with the reviewer that future work would benefit from comparison of additional non-supervised approaches for data integration, e.g. iCluster, MDI and others based on Bayesian approaches, kernel-based methods, and non-negative matrix factorization[2-4]. Discussion along these lines have been added to the main text (Lines 593-598). *3) 38% percentage of interacting proteins being in the same compartment appears quite low - is there any explaination for this? Did the authors think about integrating this data also?*

We agree with the reviewer that the 37% overlap with the hyperLOPIT dataset might appear low. However, we are actually of the opinion that for these types of studies, a 37% overlap between two complementary datasets is in fact quite high and was found to be significantly higher than comparing random proteins (p < 1.12e-203, Fishers exact test). Often independent studies, even when they utilize similar approaches, can yield relatively low overlap in the number of interactions recovered. For example, Gavin [5] and Krogan [6] both applied TAP-MS to recover 6,918 and 7,089 interactions between 1,441 and 2,694 yeast proteins respectively. Of these, only 1,172 interactions were shared (16.9% and 16.5% of the interactions in the two studies). Similarly Uetz [7] and Ito [8] both applied Y2H to yeast proteins and shared only 13.5% and 4.3% of their interactions respectively. We therefore consider our reported 38% overlap with the HyperLOPIT dataset as compelling support for the quality of our dataset.

At the same time, it should be appreciated that the hyperLOPIT dataset predicts localization and not interactions per se. Thus it is possible that our dataset may contain interactions between proteins that, while occupying different locations, nevertheless may share a point of contact (e.g. 40S ribosome and 60S ribosome, cytosol and mitochondrion membrane). Furthermore, in this study, we used protein fractions that were collected after solubilization of *Toxoplasma* cells, hence potential barriers preventing interactions between proteins have been removed. These include those associated with rhoptries, micronemes and dense granules which are secreted during host invasion and have been shown to form physiologically relevant complexes. For example the microneme and rhoptry proteins: AMA1 and RON2 respectively, have been shown to form a tight interaction that helps anchor the parasite prior to invasion [9]. We therefore looked at interactions involving proteins predicted to localize to different compartments and identified an additional 28% of interacting pairs that either share a point of contact or have the potential to interact after secretion (**Supplemental Table 8**).

The remaining (34%) pairs represent potential interactions that can be explored in future experiments. For example microneme proteins have been shown via co-IP to interact with the cytoplasmic protein, fructose-1,6-bisphosphate aldolase [10]. We now provide additional discussion on this analysis in the main text (Lines 361-380).

Finally, regarding the potential integration of the hyperLOPIT data to generate a new network, we agree this would be an interesting analysis to pursue. However, here we preferred to use the hyperLOPIT dataset as a *validation* dataset, i.e. to assess the level of performance of our network. Thus to avoid any circularity in the analysis, we could not include this dataset in our training data. We expect that future studies will build on this resource and through the integration of additional datasets, continue to improve and refine network predictions. A note to this effect has been added to the discussion (Lines 595-598). *4) It is unclear whether the experimental mass-spectrometry was replicated - as far a I understand the 480 fraction were generated once but the replicate to replicate variation wasn't examined?*

We apologize for the confusion, there were 420 and not 480 fractions. The reviewer is correct that we did not examine replicate to replicate variation. To clarify our experimental strategy the 420 fractions were derived from six different experiments - 60 fractions from each of the five beads and 120 fractions from IEX. Consistent with previous co-elution studies involving parasites [11, 12], by focusing on a diverse fractionation strategy involving six complementary experiments, we aimed to maximize the number of proteins recovered. We appreciate that the six experiments do not represent true biological replicates, however previous co-elution studies have shown replicates to be highly reproducible [13]. Furthermore through using the same lysate for each bead elution, our strategy offers the potential to capture the same protein interactions across multiple experiments. Of the 1,423 proteins identified, 187 were common to all 6 datasets. Text along these lines has been added to the main text (Lines 133-147).

*5) The FDR of ~0 doesnt really quite make sense - do the authors really believe there are no false positives with there >2,000 interactions. I think some naunce is need here too, since proteins could co-elute but not necessarily form an interaction (and visa-versa if they come apart during lysis)*

We agree with the reviewer that this does appear confusing. To clarify, we applied a score cutoff of 0.57 (corresponding to the lowest false positive rate (~0) on the ROC plot, as indicated by the ‘*’ in Figure 1D) to define a dataset of 1,541 ‘high-confidence’ interactions between 549 proteins (**Supplemental Figure 2; Supplemental Table 5**). It should be noted that while the cutoff (selected on the basis of maximizing stringency) corresponds to a FPR of 0 in the figure, this value is derived with reference to the training set and does not preclude the presence of false positives in our predictions. Text has been added to address this comment (Lines 203-205).

*Reviewer #2: review is uploaded as an attachment (240210_Review.doc)*

*In this work Swapna et al use a combination of supervised and unsupervised machine learning methodologies to integrate experimental data (biochemical co-fractionation) with functional genomics annotations (co-expression, phylogenetic profiling and domain interactions) to construct a map of protein-protein interactions in Toxoplasma gondii. The approach of combining both supervised and unsupervised methods is interesting and well-suited to a non-model organism that has limited “gold standard” training data. In general the methods are appropriate, however the rigor of the supervised model training and subsequent evaluation needs improvement. The methods is lacking some information that is necessary to understand what was done. Additionally, improving the precision of language throughout the manuscript is necessary to make it easier to follow the process of model training and data integration.*

Again we would like to thank the reviewer for their constructive comments and appreciation of our study.

*Major Comments/concerns:*

1. *Rigor of the supervised model training. The standard approach in machine learning is to split a set of “gold standard” data into training and test (validation) sets. The model is trained (often with cross validation) on the training set and then evaluated on a test set that was *not* included during training. As best I can tell, here this does not appear to be the case. My understanding is that the supervised PPI network was both trained and evaluated on a single training set. If this was the case, the model should be evaluated on a separate data set that was not included during training to have a more “fair” measure of performance. A few follow-on questions might help clarify this:*

We apologize for the confusion. The model was indeed trained through 10-fold cross-validation in which the training dataset was randomly divided into 10 sets. Each of the 10 iterations of the 10-fold cross validation used a different set as the test data, with the remaining 9 sets used to generate the training data. The ROC and AUCs (Figure 1D) represent the average performance across the 10 sets of cross validations. Text clarifying these details has been added to Methods and Results section of the main manuscript (Lines 177-188; 841-852).

a. *In figure 1, and supplementary figure 1, what data are included in the ROC and precision/recall curves that are used for network evaluation? Are these for the full training data set, or do they indicate an average performance across the 10 cross validation runs? Or were these computed on a separate test set that was not included during training?*

As detailed above, we now clarify details on network evaluation – in brief the data included in **Figure 1** and **Supplementary Figure 1** corresponds to the averaged data from 10-fold cross validation (Lines 177-188; 841-852).

b. Similarly, is the AUC of 0.806 reported in the methods (based on 10-fold cross validation) correspond to the performance across cross-validation sets, or across all the training data or across a separate hold out test set?

As explained above, we clarify that AUC of 0.806 corresponds to the average performance across 10 cross-validation sets.

2. *Model performance given inclusion of different scoring information (features). It is not clear to me how much information the experimental co-fractionation data add above the functional genomics annotations. While figure 1 and supplemental figure 1 compare the “co-elution + additional data curve” to a supervised model constructed with either co-expression or phylogenetic profiles, there is notably not a comparison to a supervised random forest classifier trained on ALL of the functional genomics data (co-expression, phylo profiles, domain-domain interactions and STRINGdb).*

We would like thank the reviewer for suggesting this additional analysis. We now include an analysis of the performance of interactions predicted using ONLY the additional functional genomics datasets (comprising of co-expression, phylogenetic profiles, domain-domain interactions; **Figure 1D & Supplemental Figure 1**). We found that the AUC while considering only functional genomics data is 0.749, lower than the AUC while using co-elution data in conjunction with functional genomics data (0.806). This emphasizes the importance of coelution data in capturing protein-protein interactions. Text along these lines has now been added to the manuscript (Lines 182-188).

3. *Methodological information. It would help to have more clarification on the process used to train the supervised model. In particular, what hyperparameters were tuned or optimized when training the Random Forest Classifier (maximum tree depth, minimum samples to split a node, others)? Also, how were the hyperparameters assigned following the 10-fold cross validation (did they take the average across the 10 training sets, or iteratively update the parameters, or something different?)*

We thank the reviewer for highlighting the importance of examining different methods. In our initial analyses, we investigated the performance of three different classifiers - RandomForest, SVM and BayesNet, using the 10-fold cross-validation framework and ultimately selecting RandomForest as it yielded the best performance in terms of AUC. The RandomForest classifier was trained using 100 trees, each constructed while considering 5 random features, at a maximum tree depth of 0 (corresponding to unlimited). The -I hyperparameter (number of trees in the Randomforest) was varied from 100 to 500, in steps of 100. However, since the AUC did not significantly vary between iterations, we chose the hyperparameter that yielded the best performance in terms of known identified complexes (100). The classifier averages the results following the 10-fold cross validation. This information has been added to the manuscript (Lines 835-839 & 841-852).

4. *Figure 1 panel A – To me, it seems that the scores (“features”) included during model training are inconsistent between this figure panel and what is listed in Supplemental Table 3. The figure suggests that 3 co-elution similarity scores were included during supervised random forest training, but from the main text and supplement it seems to me like 45 different measures of co-elution similarity were computed?*

We thank the reviewer for identifying this inconsistency. We have revised Figure 1 panel A to show the six experiments to generate coelution data. These were used to generate 45 scores which after feature selection, were reduced to 28 scores which were combined with five functional genomics scores to generate the supervised network. We have also revised the figure legend to clarify the numbers of scores used in each step (Lines 1558-1570).

*Minor Comments:*

1. *Precision of language – “scores”. Throughout the manuscript the authors refer to scores, which sometimes mean correlative measures drawn from experimental data, and sometimes mean measures drawn from functional genomics data. The functional genomics data are sometimes (though not always) called “additional data”. It would help the reader to use more descriptive, consistent naming for the scores (e.g. coelution similarity scores, functional genomics scores) throughout the paper to make it absolutely clear what is being used in the model.*

We apologize for the confusion and as suggested we have updated the language throughout the manuscript regarding the various scores used.

2. *Precision of language – model naming schemes. In some places the network constructed using the supervised and unsupervised approaches is called the “full network” and in other places “the combined network”. It would help the reader to use only one term. In my opinion “combined network” better captures the idea that the model is using information from the supervised and unsupervised approaches.*

As suggested, we have modified the manuscript to replace ‘full network’ with the term “combined network”.

3. It is not also not always clear when the authors are referring to “the combined network” and the “combined high confidence network” – for example, the introduction (p.6) states that the “pipeline yielded a network of 3,753 interactions capturing 792 proteins” while the abstract says “2,063 interactions connecting 652 proteins”. When I read this the first time I was confused by the difference in the two sets of numbers. It would be good to indicate (already in the intro) that the larger network was filtered to obtain the numbers in the abstract, and start consistently referring to the networks as the “combined model” and “combined high confidence model” from the beginning.

We apologize for the confusion. We have modified the manuscript to reflect the correct terminology throughout the manuscript, starting from the introduction. We also include the filtering step in the introduction (Lines 117-119).

4. *On p.8 the authors introduce the “scoring schemes” (“These scoring schemes were calculated individually for the 6 experiments and then once more…”). Here it would be useful to indicate that there are a total of 45 coelution correlation scores computed to give the reader a sense of the number of features.*

As suggested, we have modified the manuscript to incorporate these details in the Results section (Lines 165-166 & 177-188).

5. *Also on p.8 the authors should mention that several measures from STRING db were included as features (“functional genomics scores”) even though they did not add information (according to the red cells in table S3). At present, one needs to look in detail at the methods/supplement to learn that these were considered but did not add information.*

We apologise for the oversight and have added this information to the main text (Lines 171, 180-181).

6. *The size of the test set is described in the methods, but is sufficiently important to evaluate the results that it should be promoted to the main text (perhaps near where supplemental table 4 is introduced).*

As suggested we now mention the size of the test set in the main text (Lines 190-196).

7. *On page 9, I am confused about the “individual networks” that were constructed for the Similarity Network Fusion. Did the authors construct 7 individual networks, each based on one of the 7 scores listed (e.g. one PCCNM network, one WCC network, etc), or were the 7 networks each based on a different combination of the 7 scores (e.g. a mix of PCCNM, COEXPR-MA and MI-PresAbs)? Can the authors provide more insight into the rationale for which measures were combined/used to make the 7 individual networks?*

We apologize for the confusion concerning the generation of the SNF network. We have expanded this section to clarify the two main steps in SNF: a) construction of individual similarity networks for each of the 7 scores b) integration of different combinations of these similarity networks to provide differential weightage to the coelution, coexpression, and phylogenetic profile scores. Further, these integrated networks were generated by exploring a substantial range of the hyperparameter space. The details are added in Methods (Lines 870-893).

8. *Start of paragraph 2 on p 19 – “We exhaustively explored all possible combinations of parameters”. What parameters are being referred to here? Are these combinations of scores used to construct the networks, or model hyperparameters or something else? What was the size of the possible parameter space?*

As noted in response to point 7, we now provide additional details on the parameters explored in the construction of the networks (Lines 229-230 & 875-879).

9. *On p 10, last paragraph “Comparison of the overall supervised and unsupervised networks” – Here is the comparison between the unfiltered networks, or the filtered high-confidence networks? Again, having specific names for each network that are used consistently throughout would be a big help.*

As suggested we have clarified which networks are being compared in these analyses (unfiltered networks). Further, as suggested we now use consistent names for each of the networks in the revised manuscript (Line 248).

10. *Very minor – p. 11 “(pcc-noise model, weighted cross correlation, overall coapex score 1)” – here the authors deviate from the abbreviations used elsewhere in the paper (PCCNM, WCC)*

We apologize for the oversight. The sentence has been modified to reflect the abbreviations used throughout the manuscript (Line 257-259).

11. *p.11 “however, they are worse than that of supervised scores for PCCNM and WCC…” – I am not sure here what is being compared. Do you mean that the predicted pairwise interactions from the unsupervised model have PCCNM and WCC scores that are lower than predicted interactions in the overall supervised tree? Or do you mean that the scores of the predicted interactions from the unsupervised model are systematically lower than top hits in supervised models constructed with *only* PCCNM and WCC scores? Please define more carefully what is being compared.*

As suggested we clarify what is being compared in this section (Lines 257-260).

12. *p.11 “Based on the six scoring schemes outlined above” – I think the authors mean “six co-elution similarity scores outlined above” but the wording is somewhat ambiguous.*

We thank the reviewer for pointing this out. It was a typo and refers to the seven main scoring schemes across the coelution, coexpression and phylogenetic profile sets. We have changed the manuscript to reflect this (Lines 265-266).

13. Methods section “Capturing similarity of co-elution profiles” – p. 32. Potential typo: “Calculate similarities over 420 fractions (PCCNM_overall…)”. Within the parenthetical that lists the scoring schemes both MI-overall and MI_overall are listed, but Coapex_X is excluded?

We thank the reviewer for bringing this to our notice. MI-overall is a typo and has been deleted. However, Coapex_X is not included in this set as it refers to the score generated within each experiment, and not the one from all the experiments (Line 783).

14. *Methods section “Supervised machine learning approach” – p. 35. “61 features in total comprising of both coelution and functional dataset..” I am confused about how there are 61 features. From looking at Supplemental Table 3 I think there are 52 total features (45 coelution similarity scores and 7 functional genomics scores)? Are there additional features that I am missing?*

The reviewer is correct – there were 52 total features. We have corrected the number of features used in the supervised learning in the text, together with a breakdown of their sources (Lines 838-841).

15. *Methods – p. 36 “each of the permutations were clustered using MCL” – please define the abbreviation.*

We now spell out the acronym, which stands for Markov Cluster Algorithm (Line 884).

16. *Methods – p. 37 “For the supervised network, the network generated based on feature selection was chosen” – I am confused about what this means. Were other networks generated that were *not* based on feature selection? Or do the authors mean that they took the most informative features (colored green in supplemental table 3) and then retrained a new network after eliminating the non-informative features?*

We apologize for the confusion and have amended the text to clarify how the network was generated (Lines 912-914).

*Reviewer #3: The manuscript from Swapna et al seeks to address the knowledge gap of protein-protein interactions (PPIs) and complex formation within Toxoplasma gondii. The manuscript is generally well written, with significant amount of experimental and computational work carried out to refine and validate the interactions identified. There are a few issues that need to be addressed before publication:*

We appreciate the reviewers thoughtful comments and general recognition for our study.

*Major:
1. Page 7: The study reports 1423 unique proteins identified (supp table 2), however, over 100 proteins have been identified with only a single spectrum across 420 fractions. These are not reliable identifications and should be removed from the results.*

We apologize for the confusion. The 1423 unique proteins were those that were recognized across all fractions of which we report 162 were identified with only a single spectrum. Importantly, and as highlighted by the reviewer, for both the supervised and the unsupervised networks, we removed any pairs of interactions in which either protein of the pair had a spectral count less than 5 (406 proteins total). Thus only 1017 proteins were included in the networks and downstream analyses. We have added this additional detail to the results section (Lines 153-156, 190-196 and 240-242).

*2. Page 8: Gold standard negative interactions were generated based on differences in cellular localization. However, in page 15 and table 1, only 38% of interacting pairs shared the same localization according to hyperLOPIT. Were the rest of 62% of interacting pairs based in different localization or not identified in hyperLOPIT dataset? hyperLOPIT information should be added when generating negative controls.*

This is a very interesting point. First, and as mentioned above (reviewer 1 comment 3), we made the decision to use the hyperLOPIT dataset to assess the performance of our networks. Hence to avoid any circularity in the analysis we could not use this dataset during network generation. Future studies might wish to consider combining our co-elution data and hyperLOPIT data to generate a new network, particularly when additional validation datasets become available. However, at this stage we feel this is outside the scope of the current study. Regarding the overlap between the two datasets, as noted above, we do find that even when studies utilize the same approach, that datasets can exhibit low complementarity and indeed, we are reassured that our overlap is statistically significant. At the same time, we did revisit this analysis to further investigate correspondence between hyperLOPIT localization terms. Importantly, we note that hyperLOPIT data provides information on localization and not interactions directly. We further note that we used protein fractions that were collected after solubilization of Toxoplasma cells, hence potential barriers preventing interactions between proteins have been removed. Therefore we investigated two sets of proteins that might form putative interactions despite being allocated to different locations. These include (i) proteins that may share a point of contact (e.g. 40S ribosome and 60S ribosome, cytosol and mitochondrion membrane); and (ii) proteins associated with rhoptries, micronemes and dense granules which are secreted during host invasion and have been shown to form physiologically relevant complexes. For example the microneme and rhoptry proteins: AMA1 and RON2 respectively, have been shown to form a tight interaction that helps anchor the parasite prior to invasion [9]. From these analysis, in addition to the 38% of pairs assigned to the same compartment, and additional 28% of pairs in our network were found to either share a point of contact or have the potential to interact after secretion (**Supplemental Table 8**).

As mentioned in the response to reviewer 1, the remaining (34%) of pairs in our combined high confidence network represent hypothetical interactions that can be explored in future experiments. For example microneme proteins have been shown via co-IP to interact with the cytoplasmic protein, fructose-1,6-bisphosphate aldolase [10]. We now provide these additional details in the main text (Lines 362-380 and 595-598).

*3. Page 14: “Pearson correlation values were calculated for protein pairs across eleven time-points encompassing different lifecycle stages”. Since tachyzoite was the only lifecycle stages investigated in the study, adding expression data from other lifecycle stages would add noise to the analysis and protein interactions may not sustain across lifecycle stages, such as invasion related ones.*

As suggested, we revisited this analysis to show transcription correlation of predicted interactions utilizing three independent tachyzoites datasets spanning 10 timepoints[14, 15]. In this analysis we found that putatively interacting pairs of proteins had a significantly greater density at higher Pearson correlation values than non-interacting network proteins and a random sampling of the entire proteome. Whereas, the distribution of non-interacting network proteins was not significantly different from that of a random sampling of 10,000 proteome pairs (see Figure 2H). At the same time, the rationale for considering co-expression as a potential measure of validation is predicated on earlier studies that have shown that proteins that are co-expressed tend to interact [16]. Thus whether the proteins are expressed at a specific stage is expected to be less relevant than if two proteins exhibit similar expression patterns irrespective of stages. (Lines 343-353 and 948-972).

*Minor:
1. Page 9 and figure 1C: “the ribosome, and a snRNP complex, demonstrating its ability to capture features in the training datasets (Figure 1C…” figure 1C appear to be a wrong reference to the sentence.*

We thank the reviewer for noting this error and have removed the reference to Figure 1C accordingly (Line 210).

2. Page 12 and figure 2D: Essential proteins are highlighted as mediating more central roles in the network; however, the associated Figure 2D shows a lower betweenness centrality score for essential proteins.

The reviewer is correct and we thank them for identifying this error. The text has been modified accordingly (Line 296-300).

*3. Page 28: The 93 clusters identified should not be referred to as complexes. Comparing to experimentally validated complexes (page 16), there are many additional proteins grouped into the same cluster.*

We have modified the text as suggested (Lines 685-686).

4. Page 28: Please provide more details on how the data was made freely available on ToxoDB, which was not easily searchable.

We thank the reviewer for noting this oversight. In fact we shared a link with Omar Harb at ToxoDB at the end of 2022, however it isn’t clear what they did with the data. At the same time we realize that all of the data including the networks and cluster definitions are provided as supplemental files available under the open access terms of PLoS. Hence from the readers perspective there is no need to direct them to ToxoDB. The reference to ToxoDB has therefore being removed. That said, we are following up with Omar to find out what has happened with ToxoDB hosting our datasets.

5. Page 31: Please provide parameters used in SEQUEST searching.

We apologize for the lack of clarity, SEQUEST v2.7 was run with default parameters. We have reworded this section to include this detail (Lines 748-757).

**REFERENCES**

1.	Krishnamurthy S, Maru P, Wang Y, Bitew MA, Mukhopadhyay D, Yamaryo-Botté Y, et al. CRISPR Screens Identify Toxoplasma Genes That Determine Parasite Fitness in Interferon Gamma-Stimulated Human Cells. mBio. 2023;14(2):e0006023. Epub 2023/03/15. doi: 10.1128/mbio.00060-23. PubMed PMID: 36916910; PubMed Central PMCID: PMCPMC10128063.

2.	Shen R, Olshen AB, Ladanyi M. Integrative clustering of multiple genomic data types using a joint latent variable model with application to breast and lung cancer subtype analysis. Bioinformatics. 2009;25(22):2906-12. Epub 2009/09/18. doi: 10.1093/bioinformatics/btp543. PubMed PMID: 19759197; PubMed Central PMCID: PMCPMC2800366.

3.	Kirk P, Griffin JE, Savage RS, Ghahramani Z, Wild DL. Bayesian correlated clustering to integrate multiple datasets. Bioinformatics. 2012;28(24):3290-7. Epub 2012/10/11. doi: 10.1093/bioinformatics/bts595. PubMed PMID: 23047558; PubMed Central PMCID: PMCPMC3519452.

4.	Gligorijević V, Pržulj N. Methods for biological data integration: perspectives and challenges. J R Soc Interface. 2015;12(112). Epub 2015/10/23. doi: 10.1098/rsif.2015.0571. PubMed PMID: 26490630; PubMed Central PMCID: PMCPMC4685837.

5.	Gavin AC, Aloy P, Grandi P, Krause R, Boesche M, Marzioch M, et al. Proteome survey reveals modularity of the yeast cell machinery. Nature. 2006;440(7084):631-6. Epub 2006/01/24. doi: 10.1038/nature04532. PubMed PMID: 16429126.

6.	Krogan NJ, Cagney G, Yu H, Zhong G, Guo X, Ignatchenko A, et al. Global landscape of protein complexes in the yeast Saccharomyces cerevisiae. Nature. 2006;440(7084):637-43. Epub 2006/03/24. doi: 10.1038/nature04670. PubMed PMID: 16554755.

7.	Uetz P, Giot L, Cagney G, Mansfield TA, Judson RS, Knight JR, et al. A comprehensive analysis of protein-protein interactions in Saccharomyces cerevisiae. Nature. 2000;403(6770):623-7. Epub 2000/02/25. doi: 10.1038/35001009. PubMed PMID: 10688190.

8.	Ito T, Chiba T, Ozawa R, Yoshida M, Hattori M, Sakaki Y. A comprehensive two-hybrid analysis to explore the yeast protein interactome. Proc Natl Acad Sci U S A. 2001;98(8):4569-74. Epub 2001/04/03. doi: 10.1073/pnas.061034498. PubMed PMID: 11283351; PubMed Central PMCID: PMCPMC31875.

9.	Tonkin ML, Roques M, Lamarque MH, Pugniere M, Douguet D, Crawford J, et al. Host cell invasion by apicomplexan parasites: insights from the co-structure of AMA1 with a RON2 peptide. Science. 2011;333(6041):463-7. Epub 2011/07/23. doi: 10.1126/science.1204988. PubMed PMID: 21778402.

10.	Shen B, Sibley LD. Toxoplasma aldolase is required for metabolism but dispensable for host-cell invasion. Proc Natl Acad Sci U S A. 2014;111(9):3567-72. Epub 2014/02/20. doi: 10.1073/pnas.1315156111. PubMed PMID: 24550496; PubMed Central PMCID: PMCPMC3948255.

11.	Gazestani VH, Nikpour N, Mehta V, Najafabadi HS, Moshiri H, Jardim A, et al. A Protein Complex Map of Trypanosoma brucei. PLoS Negl Trop Dis. 2016;10(3):e0004533. Epub 2016/03/19. doi: 10.1371/journal.pntd.0004533. PubMed PMID: 26991453; PubMed Central PMCID: PMCPMC4798371.

12.	Crozier TWM, Tinti M, Wheeler RJ, Ly T, Ferguson MAJ, Lamond AI. Proteomic Analysis of the Cell Cycle of Procylic Form Trypanosoma brucei. Mol Cell Proteomics. 2018;17(6):1184-95. Epub 2018/03/21. doi: 10.1074/mcp.RA118.000650. PubMed PMID: 29555687; PubMed Central PMCID: PMCPMC5986242.

13.	Havugimana PC, Hart GT, Nepusz T, Yang H, Turinsky AL, Li Z, et al. A census of human soluble protein complexes. Cell. 2012;150(5):1068-81. Epub 2012/09/04. doi: 10.1016/j.cell.2012.08.011. PubMed PMID: 22939629; PubMed Central PMCID: PMCPMC3477804.

14.	Reid AJ, Vermont SJ, Cotton JA, Harris D, Hill-Cawthorne GA, Konen-Waisman S, et al. Comparative genomics of the apicomplexan parasites *Toxoplasma gondii* and *Neospora caninum*: Coccidia differing in host range and transmission strategy. PLoS Pathog. 2012;8(3):e1002567. Epub 2012/03/30. doi: 10.1371/journal.ppat.1002567. PubMed PMID: 22457617; PubMed Central PMCID: PMCPMC3310773.

15.	Hassan MA, Vasquez JJ, Guo-Liang C, Meissner M, Nicolai Siegel T. Comparative ribosome profiling uncovers a dominant role for translational control in Toxoplasma gondii. BMC Genomics. 2017;18(1):961. Epub 2017/12/13. doi: 10.1186/s12864-017-4362-6. PubMed PMID: 29228904; PubMed Central PMCID: PMCPMC5725899.

16.	Bhardwaj N, Lu H. Correlation between gene expression profiles and protein–protein interactions within and across genomes. Bioinformatics. 2005;21(11):2730-8. doi: 10.1093/bioinformatics/bti398.
